# Knockdown of Secernin 1 inhibit cell invasion and migration by activating the TGF-β/Smad3 pathway in oral squamous cell carcinomas

**DOI:** 10.1038/s41598-023-41504-8

**Published:** 2023-09-10

**Authors:** Li Xiao, Ting Zhang, Kaiyue Zheng, Qian Xiao, Weifang Zhang, Dandan Zhang, Dengxun Wu, Chanjuan He, Yifei Zhou, Ying Liu

**Affiliations:** 1 Affiliated Hospital of North Sichuan Medical College, Department of Stomatology, North Sichuan Medical College, Nanchong, China; 2 Department of Stomatology, Nan Chong Central Hospital, Second Clinical Medical College of North Sichuan Medical College, Nanchong, China; 3 Department of Stomatology, Lang Zhong People’s Hospital, Langzhong, China

**Keywords:** Cancer, Cell biology, Oncology

## Abstract

Secernin-1 (SCRN1) is a regulator of exocytosis in mast cells. Recently, SCRN1 was reported to be correlated with the prognosis of colorectal cancer and gastric cancer, but its functional effects on oral squamous cell carcinoma (OSCC) remain unclear. Our aim was to explore the expression pattern and the migration and invasion effects of the newly identified SCRN1 in OSCC. Western blotting (WB) was performed to measure SCRN1 expression in human OSCC tissue samples and OSCC cell lines. The effects of SCRN1 on OSCC cell proliferation, invasion and migration were analyzed by cell counting kit-8 and Transwell assays. The expression levels of TGF-β, Smad3 and phosphorylated Smad3 (p-Smad3) were measured by WB. The secretion of matrix metalloproteinase (MMP)-2 and MMP-9 was determined by the enzyme-linked immunosorbent assay. The expression of SCRN1 was significantly elevated in OSCC tissues and cell lines. SCRN1 knockdown reduced the expression of TGF-β and p-Smad3 in OSCC cells. TGF-β stimulation promoted proliferation, invasion and migration and enhanced the expression of p-Smad3 and the secretion of MMP9 in SCRN1-knockdown OSCC cell lines. Our study demonstrated that SCRN1 is upregulated in OSCC. Further analyses demonstrated that SCRN1 promotes the proliferation, invasion and migration of OSCC cells via TGF-β/Smad3 signaling.

## Introduction

Oral squamous cell carcinoma (OSCC) accounts for approximately 90% of all malignancies in the oral cavity, and it is the sixth most common cancer worldwide, with over 200,000 newly diagnosed patients each year^1,2^. Surgery remains the mainstay treatment for the management of OSCC, with adjuvant radiotherapy or chemoradiation reserved for advanced stage tumors^3^. The 5‐year survival rate of patients with OSCC in most countries is approximately 50%^4^. Approximately 30–50% of OSCC patients exhibit metastatic lymph nodes upon clinical examination, which are associated with poor outcome^5^. Due to its higher lymph node metastasis rate, low cure rate and high mortality, OSCC represents a global public health problem that poses substantial individual and socioeconomic burdens. However, the mechanism underlying the pathogenesis and metastasis of OSCC is unclear.

Secernin-1 (SCRN1) is a newly identified cytoplasmic protein that was originally shown to regulate the exocytosis of mast cells^6^. The expression of SCRN1 in gastric cancer tissues is upregulated according to cDNA microarray expression profiles, and SCRN1 is a new immunotherapeutic target^7^. A previous study suggested that upregulated SCRN1 expression is linked to poor prognosis and metastasis in colon cancer^8^, but the opposite effect has been reported in synovial sarcoma^9^. SCRN1 serves as a positive prognostic marker for synovial sarcoma, as its expression is significantly higher in patients who have survived without metastasis for least 5 years^10^. However, the expression pattern, functional effects and underlying molecular mechanism of SCRN1 in the progression of OSCC remain unknown.

Exocytosis is the process by which the contents of vesicles are released into the extracellular matrix via fusion with the cytoplasmic membrane. Cancer cells deliver surface proteins and release soluble factors (growth factors and cytokines) through exocytosis into the environment to facilitate the invasion and growth of tumors^11^. Increasing evidence demonstrates that exocytosis is associated with tumor growth, migration, and metastasis^12–14^. SCRN1 can reportedly accelerate the invasion and metastasis of colon cancer by increasing MMP-2/MMP-9 secretion^15^. The transforming growth factor (TGF)-β/Smad3 signaling pathway plays an important role in the invasion and migration of various malignancies^16–19^. Moreover, TGF-β induces the phosphorylation of Smad3 and regulates the expression of metastasis-associated genes^20^. Therefore, we hypothesized that SCRN1 promotes cell invasion and migration by activating the TGF-β/smad3 pathway in OSCC.

## Materials and methods

### Patients and specimens

This study was approved by the Ethics Committee of the North Sichuan Medical College. All specimens were collected after obtaining informed consent from patients. Primary OSCC tissue samples and matching adjacent normal mucosa specimens were collected from 15 patients undergoing surgery at North Sichuan Medical College, China. All collected tissues were stored at − 80 °C until use. The pathological or normal state of the samples was confirmed by histopathological and clinical examination.

### Tissue microarrays (TMAss)

The TMA technique was used to analyze SCRN1 expression in cancerous and adjacent tissues according to a previous method^21^. TMA sections were examined and scored by independent observers who were blinded to the clinical outcomes. Both cancerous and adjacent tissues were selected for comparative analysis. The intensity of the staining signal was measured and documented using Image‐Pro Plus 6.0 image analysis software (Media Cybernetics, Inc.). The mean densitometry of the digital image (× 500) was used to represent SCRN1 staining intensity. The signal densities in tissue areas from five randomly selected fields were determined blindly and subjected to statistical analysis.

### Cell culture and cell transfection

Human oral squamous cell carcinoma cell lines, HSC3, HSC6(human oral squamous carcinoma cells), SCC15, SCC25 (tongue squamous carcinoma cells) were obtained from GuangZhou Jennio Biotech Co.,Ltd (GuangZhou, China), UM1, UM2 (oral carcinoma cells) were obtained from ATCC (Manassas, VA, USA).Six human oral squamous cell carcinoma cell lines were cultured in Dulbecco's modified Eagle's medium (DMEM, HYCLONE, USA) supplemented with 10% fetal bovine serum, 100 U/mL penicillin, and 100 µg/mL streptomycin, and normal oral keratinocytes (NOKs) were grown in keratinocyte growth medium. All the cells were maintained at 37 °C in a 5% CO_2_ humidified incubator. Mycoplasma testing has been done for the cell lines used, the cell lines used have been authenticated.

The sh-SCRN1 plasmids and empty vector were designed and synthesized by GeneCopoeia (https://www.genecopoeia.com), and the viruses were generated using protocols from GeneCopoeia. HSC3 and SCC15 cells (2 × 10^5^/well) were seeded in 6-well plates and grown for 24 h until they reached 60–70% confluency. The cells were then transfected with diluted virus. After 12 h, the medium was replaced with fresh complement medium, and the cells were harvested after 72 h of transfection.

For the TGF-β stimulation assays, the cells were harvested after 48 h of treatment with recombinant human transforming growth factor-beta 1 (10 ng/ml).

### Western blot assay

The cells were lysed in RIPA lysis buffer (CWBIO, China) supplemented with protease inhibitors. Total proteins were fractionated using SDS-PAGE, transferred onto PVDF membranes (Millipore, USA), and examined with the corresponding primary antibodies using a chemiluminescence kit (Millipore, USA). The following primary antibodies were used in this study: anti-SCRN1 antibody (Abcam, ab105355, USA), anti-GAPDH antibody (Earthox, E021010, USA), anti-Smad3 antibody (HuaAn Biotechnology, ET1607, China), and anti-phospho-Smad3 antibody (S423/S425) (HuaAn Biotechnology, ET1609, China).

### Quantitative real-time PCR (qPCR)

Total RNA from cells was extracted using an EZNA Total RNA Kit (Omega, USA), and total RNA was reverse transcribed to cDNA in strict accordance with the manufacturer’s instructions (PrimeScript™ RT Reagent Kit, TaKaRa, Japan); the cDNA was then amplified with SYBR (Roche) by qPCR. The sequences of the primers were as follows: SCRN1 (forward: 5′-GGATGGTCTGGTGGTATTTGG-3′ and reverse: 5′-CCTTGGAACTTGGTCGATTG-3′) and GAPDH (forward: 5′-GACTCATGACCACAGTCCATGC-3′ and reverse: 5′-AGAGGCAGGGATGATGTTCTG-3′). The relative mRNA levels of SCRN1 were normalized to GAPDH reference gene expression and calculated via the 2^−∆∆CT^ method.

### Cell counting kit-8 (CCK-8) assay

The cells were seeded in 96-well plates (1000 cells/well) for the cell proliferation assay (CCK-8 kit, Yeasen, China). After culturing for 24, 48, 72 and 96 h, 10 μl of CCK-8 solution was added to each well. After adding the CCK-8 reagent, the cells were cultured for an additional 1–4 h, and the OD values were measured at 450 nm.

### Transwell migration and invasion assays

Cells (2 × 10^5^) were seeded into the upper chambers of 24-well Transwell plates containing serum-free medium. Medium supplemented with 10% FBS was added to the lower chambers. Before the Transwell invasion assays were performed, a mixture of Matrigel (BD Biosciences, USA) and medium was spread on the upper surface of the chambers and incubated at 37 °C overnight. The cells were then allowed to incubate for 24 h at 37 °C with 5% CO_2_. The cells were fixed and stained with crystal violet (0.1%). The cells that migrated from the upper chamber to the lower chamber were counted, and the average number of cells in five random regions was considered the final result.

### Enzyme-linked immunosorbent assay (ELISA)

The MMP-2/MMP-9 concentrations in the supernatants were determined by ELISA using a commercially available kit (Abcam, Cambridge, UK) in accordance with the manufacturer's specifications. The absorbance was read by a microplate reader at 450 nm. The concentration of each sample was measured based on the regression equation established using serial dilutions of standards.

### Determination of MMP2/MMP9 activity by gelatin zymography analysis

Equal amounts of total proteins were separated on 10% SDS–polyacrylamide gels containing 0.1% gelatin. The gels were washed two times with 2.5% Triton-X 100 for 20 min and incubated for 12 h at 37 °C in developing buffer consisting of 150 mM NaCl, 5 mM CaCl2 and 50 mM Tris pH 8.0. After staining with 0.5 mg/ml Coomassie Brilliant Blue R-250 in 10% acetic acid and 25% methanol for 2 h with gentle agitation, the gels were destained for an additional 2 h in 8% acetic acid and 4% methanol before being photographed.

### Statistical analysis

For continuous variables, the mean and standard deviation were used to describe baseline characteristics. Baseline characteristics between two different groups were compared by t tests for continuous variables and by chi‐square tests for categorical variables. All statistical analyses were performed by using GraphPad Prism 6.0 and Stata/MP 14.0. All the data are presented as the mean ± standard deviation (SD) from three independent experiments. A two-sided *P* ≤ 0.05 indicated statistical significance.

### Ethics approval and consent to participate

All procedures performed in the current study were approved by the Ethics Committee of North Sichuan Medical College. Written informed consent was obtained from all patients or their families.

## Results

### SCRN1 expression is significantly increased in OSCC tissues and cell lines

**Figure 1:**
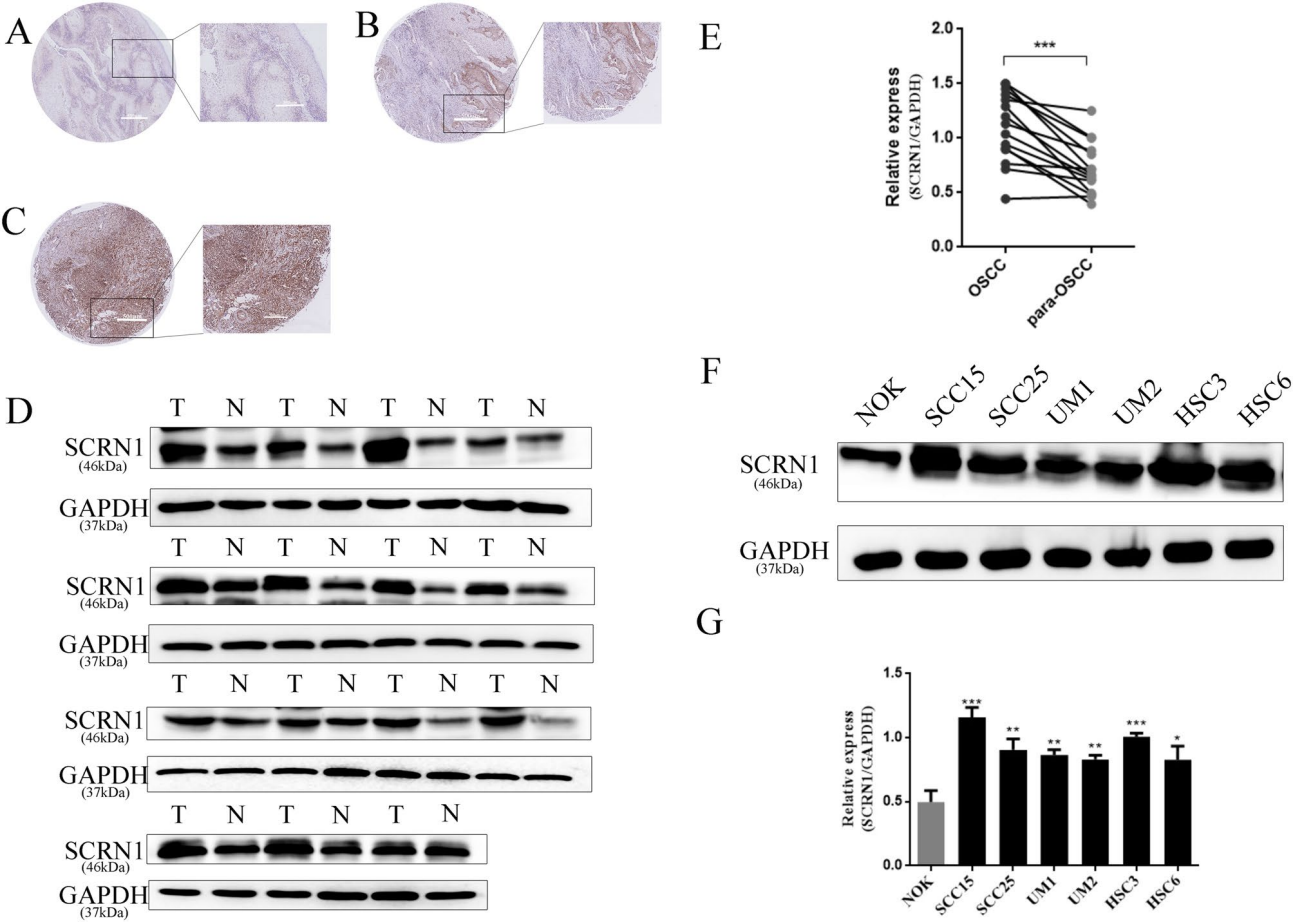
SCRN1 expression is significantly increased in OSCC tissues and cell lines. (**A**) Normal tissue was negative for SCRN1 staining (× 500). (**B**) Weak expression of SCRN1 was defined as low expression in OSCC (× 500). (**C**) Strong expression of SCRN1 with cytoplasmic and membranous staining was defined as high expression in OSCC (× 500). (**D**, **E**) The expression levels of SCRN1 were measured in 15 paired OSCC tissues and adjacent normal mucosa by WB. The data are presented as the mean ± SD. ***p < 0.001. (**F, G**) The expression levels of SCRN1 in the HSC3, HSC6, SCC15, SCC25, UM1 and UM2 cell lines were examined by WB. Significant differences were determined using t test. The data are presented as the mean ± SD of three independent experiments. *p < 0.05, **p < 0.01 and ***p < 0.001.

Tissues from 110 patients with OSCC were subjected to immunohistochemistry (IHC) analysis. As shown in Fig. 1A–C, SCRN1 expression was low in normal tissues (Fig. 1A) and low (Fig. 1B) and high (Fig. 1C) in tumor tissues. According to the level of SCRN1 expression, we divided these patient specimens into two groups: high SCRN1 expression and low SCRN1 expression. The comparison of the clinicopathological characteristics between the two groups is shown in Table 1. The distributions of OSCC patients in the low and high expression groups were significantly different in regards to the tumor stage, nodal stage, and clinical TNM stage (*p* < 0.05), but no significant differences in sex, smoking status, drinking status, tumor differentiation, primary lesion, surgery status, radiotherapy history, or chemotherapy history were observed (*p* > 0.05). To further quantify the expression level of SCRN1 in OSCC, SCRN1 protein expression was compared between 15 cancerous tissues and adjacent normal mucosal tissues by WB. Among the 15 OSCC samples, 12 exhibited higher SCRN1 expression than that in the paired normal mucosa, and the difference between the OSCC tissue and adjacent normal mucosa was statistically significant (Fig. 1D–E, *P* < 0.001). Furthermore, the SCRN1 expression level was analyzed by WB in OSCC cell lines, including HSC3, HSC6, SCC15, SCC25, UM1 and UM2 (Fig. 1F, G). SCRN1 expression was significantly upregulated in OSCC cells compared to NOKs (*P* < 0.05). The highest expression level was presented in the HSC3 and SCC15 cells. Therefore, the HSC3 and SCC15 cell lines were chosen for functional assays. Taken together, these results showed that the high expression of SCRN1 was positively correlated with the OSCC stage and distant metastasis; for the first time, SCRN1 was shown to be highly expressed in OSCC tissues and 6 OSCC cell lines compared to the controls. Therefore, the increased expression of OSCC may be closely related to the development of OSCC.

**Table 1:** Clinicopathological characteristics of 110 OSCC patients according to SCRN1 expression.

Characteristic	Total (N = 110)	Low expression (N = 43) number (%)	High expression (N = 67) Number (%)	*P* values*
Gender				
Male	69 (62.7)	28 (65.1)	41 (61.2)	0.6924
Female	41 (37.3)	15 (44.9)	26 (38.8)	
Smoking status				
Never	49 (44.5)	23 (53.5)	26 (38.8)	0.1692
Ever	61 (55.5)	20 (46.5)	41 (61.2)	
Drinking status				
Never	40 (36.4)	19 (44.2)	21 (31.3)	0.2233
Ever	70 (63.6)	24 (55.8)	46 (68.7)	
Tumor differentiation				
Poor	35 (31.8)	10 (23.3)	25 (37.3)	0.1452
Well-moderate	75 (68.2)	33 (76.7)	42 (62.7)	
Primary site				
Ventral tongue	32 (29.1)	12 (29.1)	20 (29.1)	0.6236
Buccal mucosa	44 (40.0)	20 (40.0)	24 (40.0)	
Gingiva	21 (19.1)	6 (19.1)	15 (19.1)	
Others^a^	13 (11.8)	5 (11.8)	8 (11.8)	
Tumor stage				
T1/T2	68 (61.8)	33 (76.7)	35 (52.2)	0.0153
T3/T4	42 (38.2)	10 (23.3)	32 (47.8)	
Nodal stage				
N0	72 (65.5)	34 (79.1)	38 (56.7)	0.0233
N1-N3	38 (34.5)	9 (20.9)	29 (43.3)	
Clinical TNM stage				
I/II	58 (52.7)	29 (67.4)	29 (43.3)	0.0186
III/IV	52 (47.3)	14 (32.6)	38 (56.7)	
Surgery type				
Simple LR	49 (44.5)	21 (48.8)	28 (41.8)	0.5563
Other	61 (55.5)	22 (51.2)	39 (58.2)	
Radiotherapy history				
Yes	30 (27.3)	9 (20.9)	21 (31.3)	0.2767
No	80 (72.6)	34 (79.1)	46 (68.7)	
Chemotherapy				
Yes	65 (36.4)	30 (44.5)	35 (36.4)	0.0765
No	45 (63.6)	13 (55.5)	32 (63.6)	

### Knockdown of SCRN1 suppresses the proliferation, invasion and migration of OSCC cell lines

**Figure 2:**
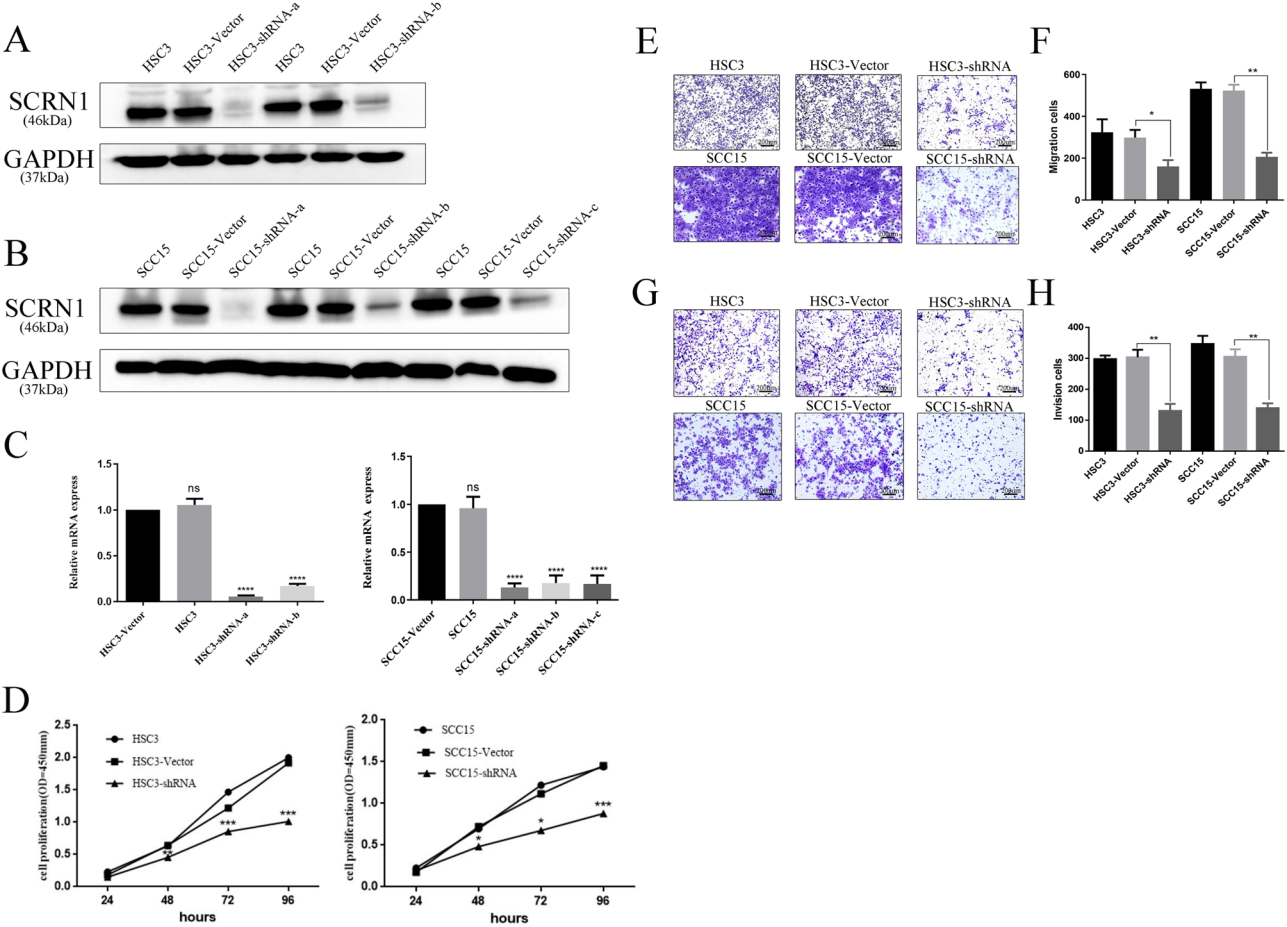
Knockdown of SCRN1 suppresses proliferation, invasion and migration in OSCC cell lines. (**A**) The protein expression levels of SCRN1 in HSC3 cell transfected with three short-hairpin RNAs, namely, shRNA-a, shRNA-b and shRNA-c, were evaluated by WB. (**B**) The protein expression levels of SCRN1 in SCC15 cell transfected with three short-hairpin RNAs, namely, shRNA-a, shRNA-b and shRNA-c, were evaluated by WB. (**C**) The mRNA expression levels of SCRN1 in HSC3 and SCC15 cells transfected with three short-hairpin RNAs, namely, shRNA-a, shRNA-b and shRNA-c, were evaluated by qPCR. (**D**) SCRN1 knockdown suppressed HSC3 and SCC15 cell proliferation, as determined by the CCK-8 assay. (**E, F**) Transwell migration assays were performed to assess and quantify the migration of SCRN1-knockdown and control HSC3 and SCC15 cells. (G-H) Transwell migration assays were performed to assess and quantify the invasion of SCRN1-knockdown HSC3 and SCC15 cells and control cells. The data are presented as the mean ± SD of three independent experiments. **p < 0.01, ***p < 0.001 and ****p < 0.0001.

SCRN1 promotes exocytosis in cells, which regulates the secretion of several cytokines and proteins related to metastasis such as MMP-2/MMP-9, thus promoting the metastasis of tumors^15^. Therefore, we hypothesized that SCRN1 plays a role in the migration and invasion of OSCC. To investigate the effects of SCRN1 on OSCC proliferation, invasion and migration, we selected two OSCC cell lines, HSC3 and SCC15, due to their relatively high SCRN1 expression. To establish SCRN1 stable knockdown cell lines, WB and qPCR assays were performed to identify the optimal short hairpin RNA (shRNA) among three selected putative shRNAs (shRNA-a, shRNA-b, and shRNA-c) (Fig. 2A–C). When HSC3 cells were transfected with shRNA-c, the majority of the cells died. We repeated the experiment three times, and the results remained consistent. The results indicated that shRNA-a had the highest knockdown efficiency and was therefore used to produce the knockdown cell line.

The CCK-8 proliferation assay revealed that the proliferation capacity of the OSCC cells was diminished after SCRN1 knockdown (Fig. 2D). In the Transwell migration assay, SCRN1 knockdown significantly reduced the migration ability of HSC3 cells compared to that of control cells (*P* < 0.05). Similar results were observed in SCC15 cells (*P* < 0.01) (Fig. 2E, F). The Transwell invasion assay demonstrated that the number of invasive SCRN1 knockdown HSC3 cells was almost three times lower than that of vector cells (*p* < 0.01) (Fig. 2G, H). In SCC15 cells, similar effects were observed (*p* < 0.01). Thus, these results showed that SCRN1 promotes cancer cell proliferation, invasion and migration in OSCC.

### SCRN1 promotes cell invasion and migration by activating the TGF-β/Smad3 pathway

**Figure 3:**
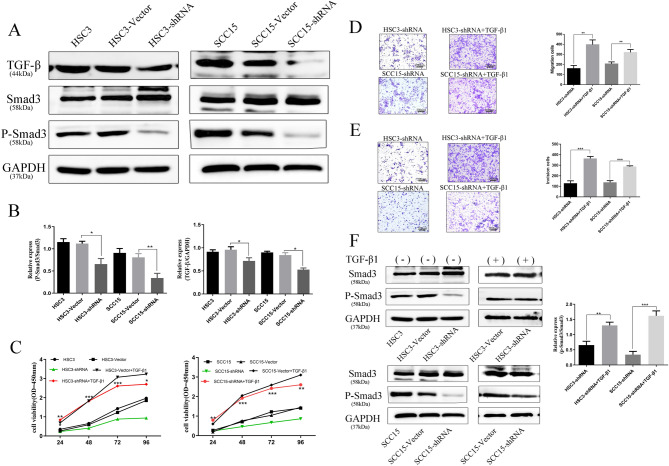
SCRN1 promotes cell invasion and migration by activating the TGF-β/Smad3 pathway. (**A**) The protein expression levels of TGF-β, Smad3 and p-Smad3 in HSC3 and SCC15 cells were evaluated by WB. (**B**) The levels of TGF-β and p-Smad3 in SCRN1-knockdown HSC3 and SCC15 cells and control cells were determined by WB. The ratios of p-Smad3 to the total levels were calculated based on the densities of the respective bands. The data are presented as the mean ± SD of three independent experiments. **p < 0.01. (**C**) After 48 h of treatment with TGF-β, SCRN1-knockdown HSC3 and SCC15 cell proliferation was assessed by the CCK-8 assay. Compared with the HSC3-shRNA group (Green), TGF-β stimulation promoted the proliferation of OSCC cell lines in the HSC3-shRNA + TGF-β group (Red). (**D**) After 48 h of treatment with TGF-β, Transwell invasion and migration assays were performed to assess and quantify migration in SCRN1-knockdown HSC3 and SCC15 cells and control cells. (**E**) After 48 h of treatment with TGF-β, Transwell invasion and migration assays were performed to assess and quantify invasion in SCRN1-knockdown HSC3 and SCC15 cells and control cells. (**F**) After 48 h of treatment with TGF-β, the levels of p-Smad3 in HSC3 and SCC15 cells were determined by WB. The ratios of p-Smad3 to the total levels were calculated based on the densities of the respective bands. The data are presented as the mean ± SD of three independent experiments. **p < 0.01.

We further aimed to elucidate the potential molecular mechanisms by which SCRN1 regulates the proliferation, migration and invasion of OSCC cells. Since accumulating studies have confirmed that TGF-β/Smad3 signaling plays extensive regulatory roles in the development and progression of multiple types of cancers, we next conducted Western blot assays to assess the effects of SCRN1 knockdown on the activity of TGF-β/Smad3 signaling. Knockdown of SCRN1 reduced the TGF-β and P-Smad3 levels in HSC3 cells but did not change that of Smad3 in HSC3 cells (Fig. 3A, B). Similar results were observed for SCC15 cells (Fig. 3A, B).

To further confirm that SCRN1 functions a tumor promoter in OSCC by regulating the TGF-β/Smad3 pathway, we next assessed the effects of TGF-β on OSCC cell proliferation, migration and invasion. Then, TGF-β was used to stimulate SCRN1-knockdown OSCC cell lines. Compared with the HSC3-shRNA group, TGF-β stimulation promoted the proliferation of OSCC cell lines in the HSC3-shRNA + TGF-β group (Fig. 3C), suggesting that TGF-β treatment reversed the reduction in OSCC proliferation caused by SCRN1 knockdown. Similar results were observed for SCC15 cells (Fig. 3C). TGF-β treatment promoted the migration and invasion of OSCC cell lines in the HSC3-shRNA + TGF-β group compared to OSCC cell lines in the HSC3-shRNA group (Fig. 3D, E). Similar results were obtained for the SCC15 cell line (Fig. 3D, E).

We then detected the expression of Smad3 and P-Smad3 in SCRN1-knockdown OSCC cells after TGF-β treatment by WB. TGF-β stimulation of the stable knockdown SCRN1 cells upregulated P-Smad3 expression (Fig. 3F), suggesting that the TGF-β signaling pathway and its downstream signaling molecules were partially blocked by SCRN1 knockdown. Therefore, these results indicate that SCRN1 promotes cell invasion and migration by activating the TGF-β/Smad3 pathway in OSCC.

### SCRN1 enhances MMP-9 exocytosis by activating the TGF-β/Smad3 pathway

**Figure 4:**
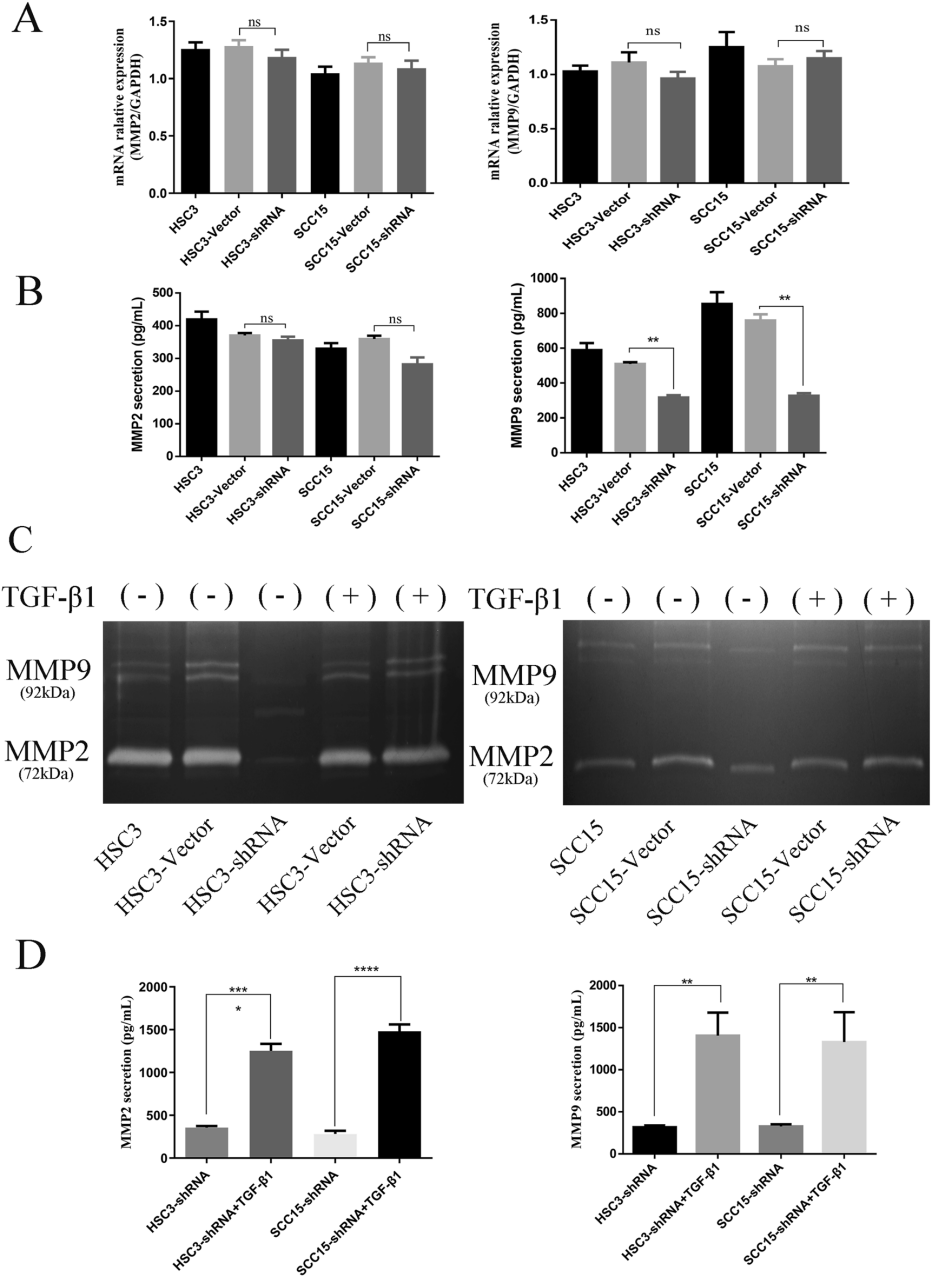
SCRN1 enhances MMP-9 exocytosis by activating the TGF-β/Smad3 pathway. (**A**) qPCR revealed that no significant difference in MMP-2/9 mRNA between control cells and SCNR1-knockdown cells. The data are presented as the mean ± SD, P > 0.05 and ns = no significant difference. (**B**) MMP-2 and MMP-9 protein secretion from SCRN1 knockdown SCC15 and HSC3 cells as determined by ELISA. The data are presented as the mean ± SD of three independent experiments. **p < 0.01. (**C**) Gelatinase activity as determined by zymogram densitometric analysis. The data are presented as the mean ± SD of three independent experiments. (**D**) After 48 h of treatment with TGF-β, MMP-2 and MMP-9 protein from SCRN1-knockdown SCC15 and HSC3 cells was detected by ELISA. The data are presented as the mean ± SD of three independent experiments. **p < 0.01.

MMPs, particularly MMP-2 and MMP-9 gelatinases, mediate extracellular matrix degradation, thereby facilitating the invasion and spread of OSCC cells^22^. Therefore, we further verified that SCRN1 contributes to the invasion and migration of OSCC cells. qPCR analysis was performed to determine the effect of SCRN1 on MMP-2/9 gene expression. No decrease in MMP-2/9 mRNA expression was observed in SCRN1-knockdown HSC3 cells or SCRN1-knockdown SCC15 cells compared to vector cells (Fig. 4A). MMP-2/9 secretion was investigated in control and SCRN1-knockdown OSCC cells using ELISA. Significant inhibition of MMP-9 secretion was observed in SCRN1-knockdown SCC15 and HSC3 cells compared to vector cells (*p* < 0.01) (Fig. 4B), but no obvious change in MMP-2 secretion was observed (*p* > 0.05).

To clearly show that SCRN1 affects extracellular matrix degradation, we performed zymographic analysis of MMP2/MMP9 activity. After irradiation, decreased gelatinolytic activity was observed in SCRN1-knockdown OSCC cell lines compared with that in the control cell lines (Fig. 4C). TGF-β stimulation of the stable knockdown SCRN1 cells upregulated gelatinolytic activity of MMP2/MMP9 activity (Fig. 4C).

A previous study indicated that TGF-β promotes tumor invasion and metastasis by activating MMPs^23^. Our results demonstrated that SCRN1 promotes tumor invasion and migration by activating the TGF-β signaling pathway. Therefore, we investigated whether SCRN1 promotes MMP-9 secretion and TGF-β signaling pathway activation. We found that upon TGF-β stimulation, MMP-9 secretion was increased in the OSCC-shRNA + TGF-β group compared with the SCRN1-knockdown OSCC group (p < 0.01) (Fig. 4D), similar results were observed for MMP-2 secretion (Fig. 4D). These results indicated that SCRN1 enhances the exocytosis of the MMP-9 protein by activating the TGF-β signaling pathway but has little impact on MMP-2 exocytosis.

## Discussion

Despite recent advances in treatment modalities, including surgery, chemotherapy, biological therapy, and radiotherapy, the overall survival of OSCC patients has remained poor over the past few years^24^. Local invasion and lymph node metastasis are still important reasons for the poor prognosis of OSCC^25^. Recently, the identification of key molecular alterations in cancer has resulted in major advances in diagnosis and targeted therapies with validated biomarkers. Therefore, the discovery of novel biomarkers for targeted therapeutics to improve the clinical survival rate of OSCC patients is urgently needed. Our study focused on the newly identified protein, SCRN1. Advanced T stage, LNM, distant metastasis, and poor differentiation are known as the main factors affecting the progression and prognosis of patients with OSCC. We found that high SCRN1 expression was correlated with the tumor stage, nodal stage, and clinical TNM stage. Moreover, SCRN was highly expressed in 15 OSCC tissue specimens and 6 OSCC cell lines, which was consistent with its expression in gastric cancer and colorectal cancer^7,8^.

SCRN1 regulates mast cell exocytosis through a well-defined mechanism. Exocytosis is a process by which cells transport and release secretory products through the cytoplasm to the cell membrane, and this process promotes tumor growth, metastasis and invasion. Lin et al. ^15^ reported that SCRN1 expression is upregulated in colorectal cancer and that its overexpression promotes the proliferation and invasion of colorectal cancer cells. However, the function of SCRN1 in OSCC remains unknown. In this study, we performed CCK-8 and Transwell assays to investigate the functions of SCRN1 in OSCC cells (HSC3/SCC15), and we found that knockdown of SCRN1 suppressed OSCC cell proliferation, migration and invasion.

Many classical and critical signaling pathways, such as Wnt/β-catenin, TGF-β and PI3K/Akt/mTOR, are known to be involved in the regulation of tumor growth and progression^26–28^. Despite the well‐known TGF‐β suppressor effect during the early stages of tumor development, there is strong evidence that TGF‐β might later contribute to promoting proliferation, invasion, angiogenesis, and oncogenesis in advanced cancer^29^. Increasing evidence has confirmed that many novel markers promote tumor progression by activating the TGF‐β pathway^30,31^. Recently, increasing numbers of studies have reported that exocytosis-associated proteins may regulate the activation of the TGF‐β pathway, thereby influencing the progression of tumors^32,33^. However, the association between SCRN1 and the TGF‐β/Smad3 pathway remains largely unclear. To explore the potential mechanism by which SCRN1 promotes OSCC cell proliferation, invasion and migration, we investigated its influence on the TGF‐β/Smad3 pathway, which is the most common TGF pathway. The WB results indicated that knockdown of SCRN1 decreased the p-Smad3 levels. Moreover, TGF‐β treatment enhanced the proliferation, invasion and migration of SCRN1-knockdown OSCC cell lines and upregulated their levels of p-Smad3. TGF‐β treatment reversed the decreases in the proliferation, invasion and migration of tumor cells induced by SCRN1 knockdown as well as the decrease in p-SMad3 expression in OSCC cell lines, suggesting that SCRN1 acts promotes OSCC by modulating the TGF‐β/Smad3 pathway.

MMP exocytosis, especially MMP-2/9 exocytosis, is essential for tumor cell invasion and metastasis ^34^. Because SCRN1 regulates exocytosis in other cell types, we speculated that it may increase the secretion of MMP-2/9 to promote OSCC cell invasion and migration. The results revealed no significant effect of SCRN1 on the mRNA expression of MMP-2/9 in OSCC cell lines as determined by qPCR. ELISAs demonstrated that MMP-9 secretion was suppressed in SCRN1 knockdown cells but that MMP-2 secretion was not significantly changed. In addition, after TGF‐β stimulation, the secretion of MMP-9 was increased in SCRN1-knockdown OSCC cell lines, indicating that SCRN1 enhances MMP-9 exocytosis by activating the TGF-β/Smad3 pathway. These results indicated that SCRN1 promotes the proliferation, migration and invasion of OSCC cells by enhancing the secretion of MMP-9. Further studies are needed to determine whether SCRN1 facilitates the proliferation, migration and invasion of OSCC cells by targeting the TGF-β-Smad3-MMP-9 pathway.

In summary, our data provided evidence, for the first time, that high expression of SCRN1 is correlated with the tumor stage, nodal stage, and clinical TNM stage. SCRN1 was shown to be highly expressed in OSCC tissue, and SCRN1 knockdown inhibited OSCC cell proliferation, invasion and migration. Moreover, our results showed that SCRN1 may contribute to the progression of OSCC by enhancing the secretion of MMP-9 and activating the TGF‐β/Smad3 pathway. Activation of TGF‐β/Smad3 and MMP signaling has been frequently associated with OSCC progression. In our study, both pathways were shown to be involved in the oncogenic functions of SCRN1.

### Supplementary Information

Supplementary Information 1.

Supplementary Information 2.

## Data Availability

The datasets used and/or analyzed during the current study are available from the corresponding author upon reasonable request.
