# Supplementary material for: Knockdown of Secernin 1 inhibit cell invasion and migration by activating the TGF-β/Smad3 pathway in oral squamous cell carcinomas
Source: Sci Rep. 2023 Sep 10;13:14922. doi: 10.1038/s41598-023-41504-8 (PMC10493221; doi:10.1038/s41598-023-41504-8)
Supplement: Supplementary file 1 — Supplementary Information 1. [file 41598_2023_41504_MOESM1_ESM.docx]

maintenance and culture of cell lines

An in-depth description of basic culture parameters in accordance with GCCP is important for replication of the work and interpretation of experimental data.

1. the list of reagents

Dulbecco's modified Eagle's medium (HYCLONE, USA)

Fetal bovine serum (HYCLONE, USA)

Penicillin (HYCLONE, USA)

Streptomycin (HYCLONE, USA)

Phosphate buffer solution (HYCLONE, USA)

Trypsin solution (HYCLONE, USA)

Serum-free cell freeze Medium (Biological Industries, Israel)

2) the step-wise process and incubation conditions required

2.1 Cell culture

Human normal oral keratinocyte cell lines and oral squamous cell cancer cell lines were cultured in Dulbecco's modified Eagle's medium supplemented with 10% fetal bovine serum, 100 U/mL penicillin, and 100 µg/mL streptomycin. When the cell growth reached the fusion rate of 70%-80%, the cells were washed with sterile PBS twice, then digested with trypsin solution for 3min, centrifuged, and the collected cells were used for passage or experiment.

2.2 Cell passage

When the cells covered the petri dish nearly 80%, the old medium was removed, and after 2 times of cleaning with PBS, 1mL of trypsin rewarmed at 37℃ was added, and then placed in the incubator at 37℃ for 1 ~ 2min, gently shaken, and observed under an optical microscope, it was found that a large number of cells became round in shape and had enhanced refraction. The complete cell medium was added to terminate digestion. The bottom of the cell culture dish was blown repeatedly with a pipette gun, and the cells were completely collected and centrifuged in a 15mL centrifuge tube at 1200rpm for 3min. Remove the supernatant, leave the cell precipitate, add into the fresh and complete medium, mix well, subpackage and pass in a new culture dish for further culture.

2.3 Cell count

The cover glass is covered over the clean cell counting plate, 10μL of mixed cell suspension is absorbed and dripped on the edge of the cover glass to make it evenly distributed on the counting plate to avoid bubbles. The cells in four large squares were counted under an optical microscope. Cell density is calculated as follows: Number of cells /mL= (total /4) ×10^4^/mL

2.4 Cell resuscitation

Remove the frozen storage tube from the liquid nitrogen tank and quickly melt its contents in a 37℃ water bath (within 1min) to avoid contamination of the frozen storage tube mouth. The cell suspension was absorbed and quickly added into a 15mL aseptic centrifuge tube pre-loaded with 10mL complete cell medium, centrifuged at 1200rpm for 3min, the supernatant was discarded, fresh cell medium was added, and then transferred into a new cell culture dish after mixing.

2.5 Cell cryopreservation

When the cell density reaches 80 ~ 90%, the cells should be frozen to avoid contact inhibition. The cell cryopreservation solution was added to the cell precipitation after trypsin digestion, mixed and transferred quickly to the cryopreservation tube, sealed with a film, placed in a -80℃ refrigerator overnight, stored in a liquid nitrogen tank, and used within one year.
