# Supplementary material for: Knockdown of Secernin 1 inhibit cell invasion and migration by activating the TGF-β/Smad3 pathway in oral squamous cell carcinomas
Source: Sci Rep. 2023 Sep 10;13:14922. doi: 10.1038/s41598-023-41504-8 (PMC10493221; doi:10.1038/s41598-023-41504-8)
Supplement: Supplementary file 2 — Supplementary Information 2. [file 41598_2023_41504_MOESM2_ESM.pptx]

## Slide 1
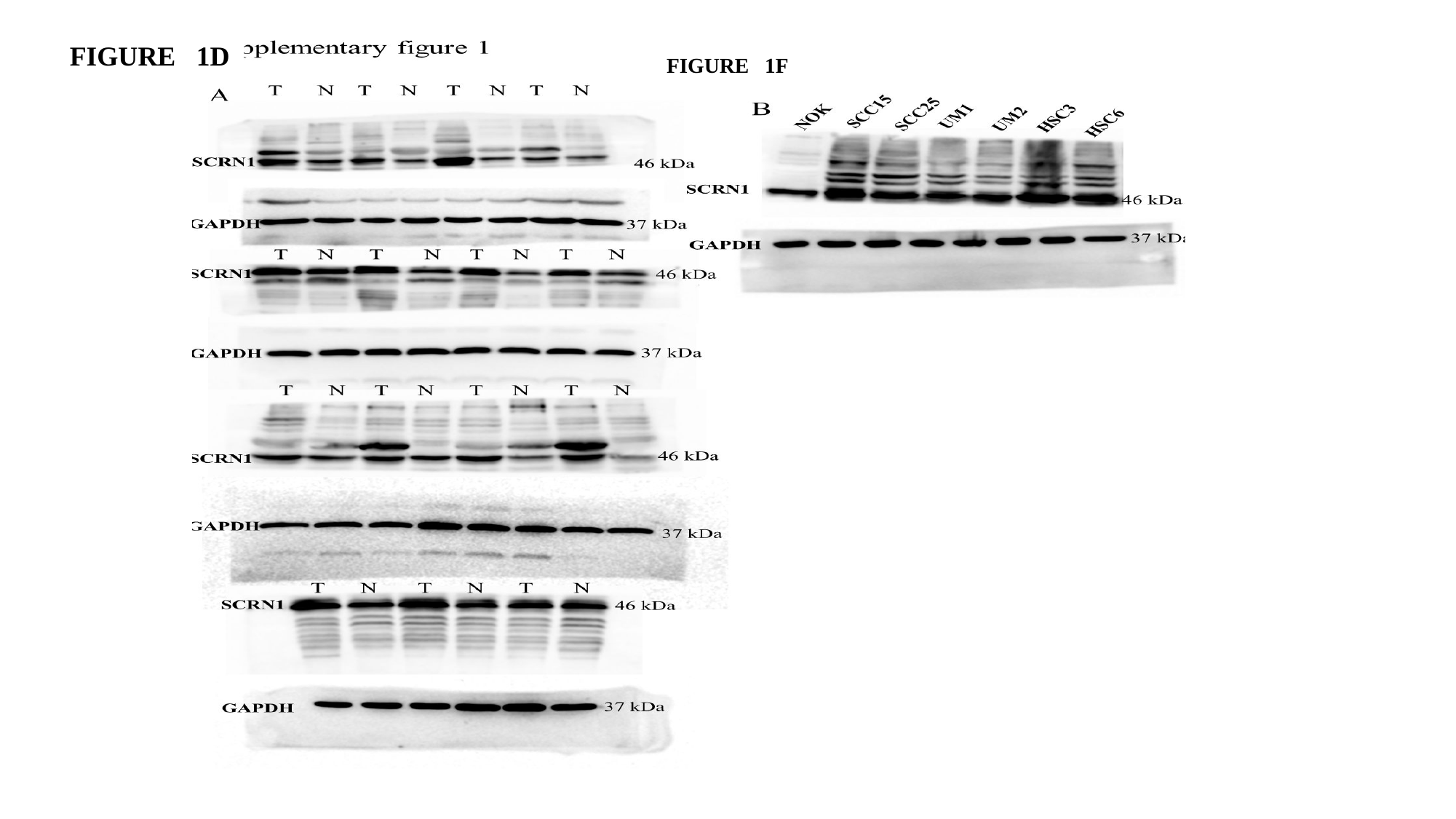

FIGURE 1D
FIGURE 1F

## Slide 2
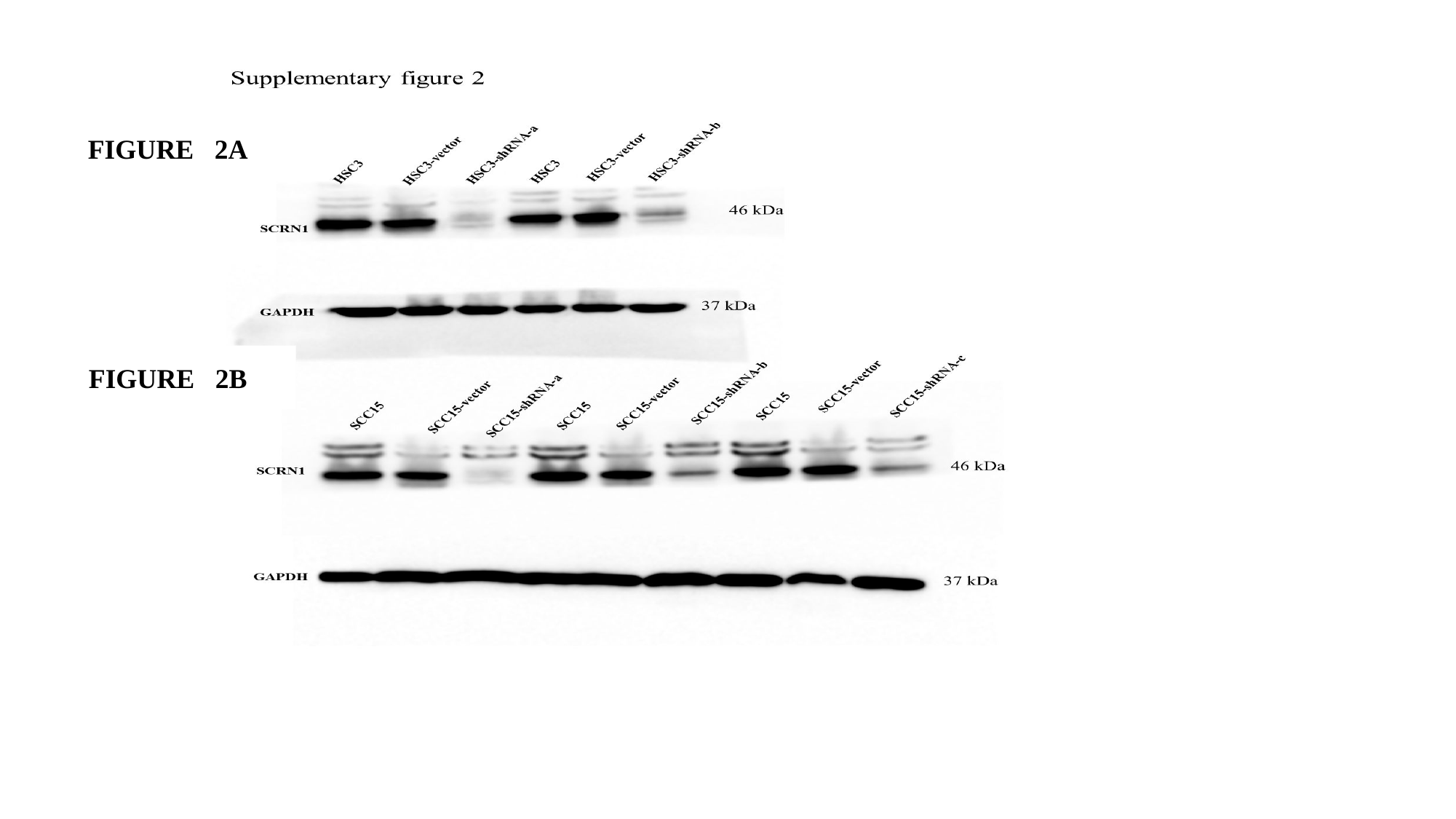

FIGURE 2A
FIGURE 2B

## Slide 3
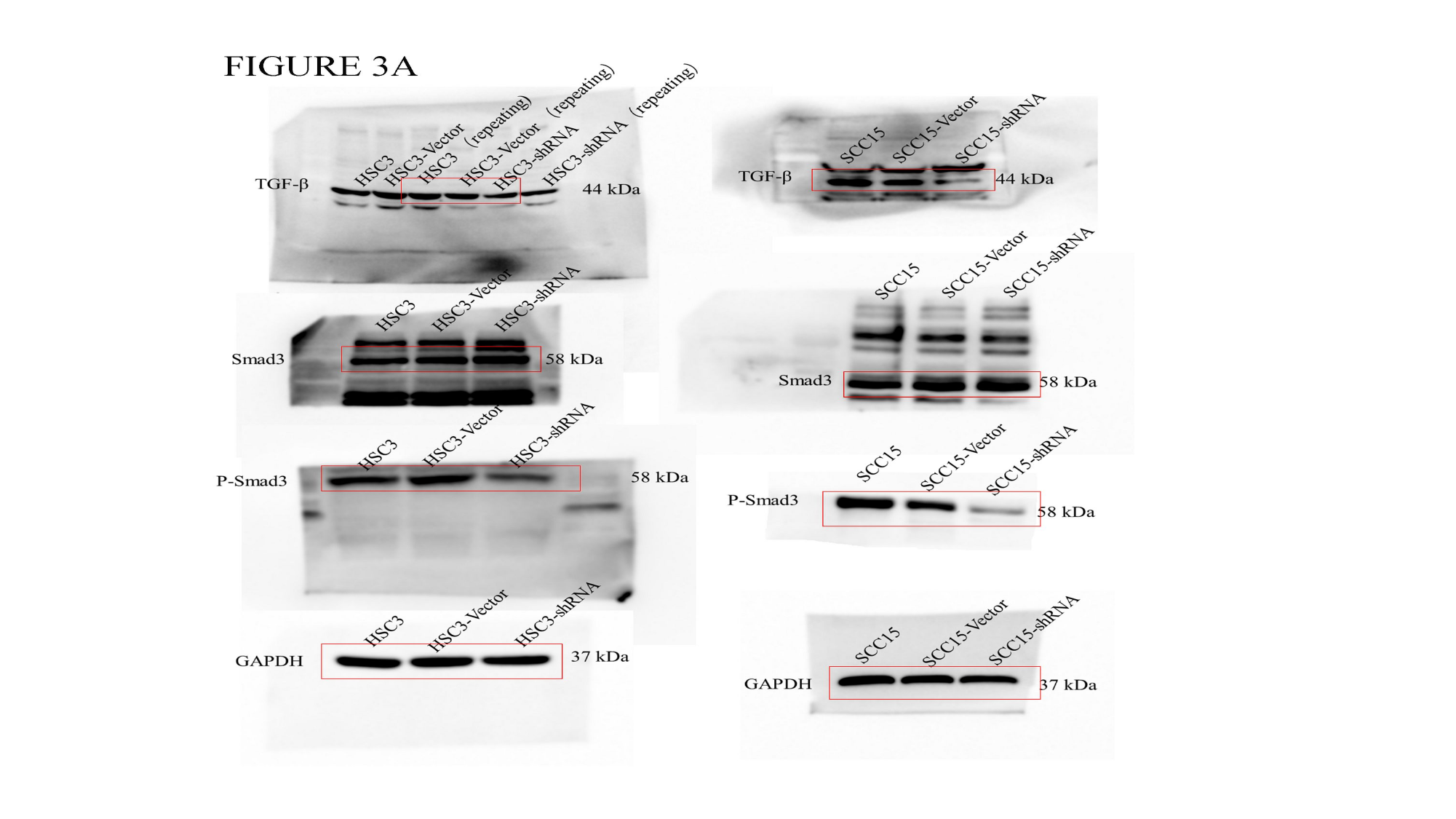

## Slide 4
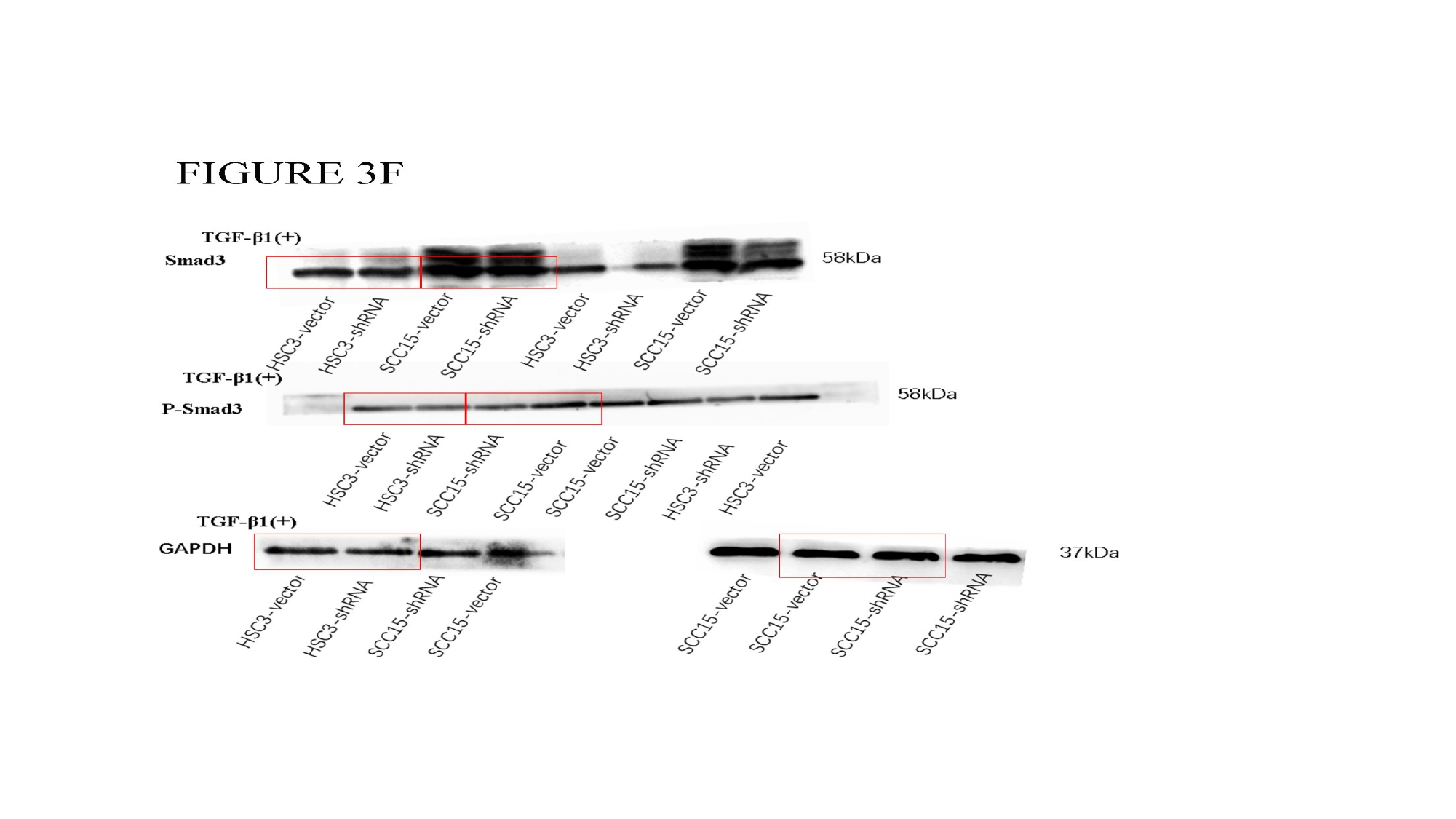

## Slide 5
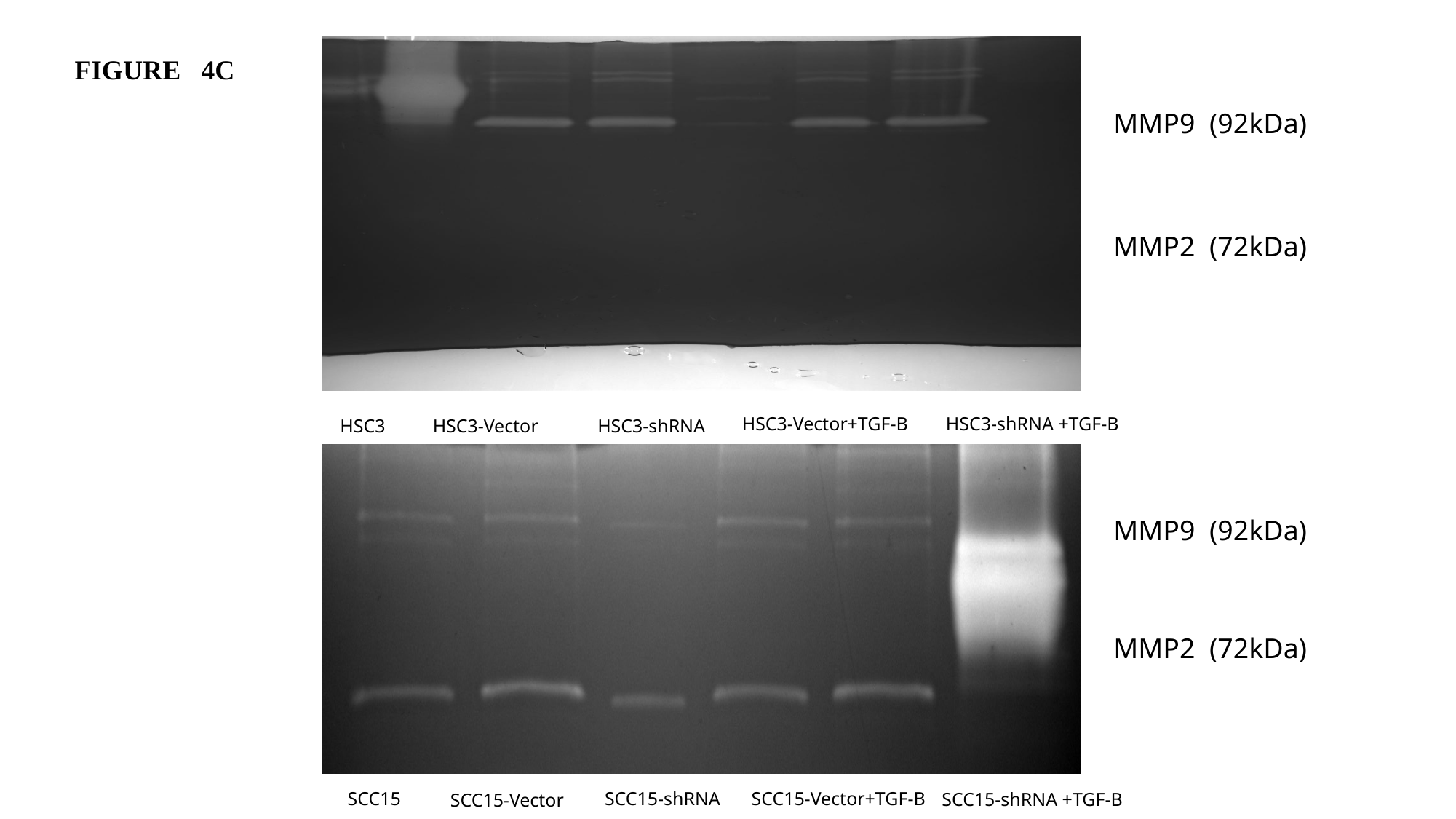

FIGURE 4C
MMP9 (92kDa)
MMP2 (72kDa)
HSC3-Vector+TGF-B
HSC3-shRNA +TGF-B
HSC3
HSC3-Vector
HSC3-shRNA
MMP9 (92kDa)
MMP2 (72kDa)
SCC15-Vector+TGF-B
SCC15-shRNA
SCC15
SCC15-shRNA +TGF-B
SCC15-Vector
